# Risk factors and cancer recurrence associated with postoperative complications after thoracoscopic lobectomy for clinical stage I non‐small cell lung cancer

**DOI:** 10.1111/1759-7714.13173

**Published:** 2019-08-21

**Authors:** Takeo Nakada, Yuki Noda, Daiki Kato, Takamasa Shibasaki, Shohei Mori, Hisatoshi Asano, Hideki Matsudaira, Jun Hirano, Makoto Odaka, Takashi Ohtsuka

**Affiliations:** ^1^ The Jikei University School of Medicine, Department of Surgery, Division of Thoracic Surgery Tokyo Japan

**Keywords:** Lung cancer, postoperative complication, risk factor, sarcopenia, thoracoscopic surgery

## Abstract

**Background:**

Minimally invasive thoracoscopic lobectomy is the recommended surgery for clinical stage I non‐small cell lung cancer (NSCLC). The purpose of this study was to identify the risk factors, including sarcopenia, for postoperative complications in patients undergoing a complete single‐lobe thoracoscopic lobectomy for clinical stage I NSCLC, as well as the impact of complications on disease‐free survival.

**Methods:**

We retrospectively investigated 173 patients with pathologically‐diagnosed NSCLC who underwent curative thoracoscopic lobectomies between April 2013 and March 2018. Sarcopenia was assessed using the psoas muscle index calculated from preoperative computed tomography images at the third lumbar vertebral level.

**Results:**

Complications developed in 38 (22%) patients, including 21 with prolonged air leak. In univariate analysis, the significant risk factors for complications were advanced age, male sex, higher Charlson Comorbidity Index (CCI) score, lower cholinesterase, lower albumin, higher creatinine level, pleural adhesion, operative time ≥ five hours, nonadenocarcinoma cancer, and larger tumor size. Multivariate analysis showed that age ≥ 75 years (*P* = 0.002) and pleural adhesion *(P* = 0.026) were significant independent risk factors for complications. Compared with the patient group without complications, postoperative complications were independently associated with shorter disease‐free survival *(P* = 0.01).

**Conclusions:**

Advanced age and pleural adhesion were independent risk factors for complications after complete single‐lobe thoracoscopic lobectomies for clinical stage I NSCLC, and postoperative complications were statistically associated with poor prognosis. Surgical teams should ensure an experienced surgeon leads the operation for patients at higher risk to avoid prolonged postoperative hospitalization and a possible poor prognosis.

## Key points

### Significant findings of the study

Advanced age and pleural adhesion were independent risk factors for complications after complete single‐lobe thoracoscopic lobectomies for clinical stage I NSCLC. Sarcopenia was not a risk factor for postoperative complications or shorter DFS. Postoperative complications were statistically associated with poor prognosis.

### What this study adds

Many other risk factors had been significantly associated with postoperative complications after thoracotomy. Nowadays, surgeons have questioned “high risk” for lung resection patients using minimally invasive thoracoscopic lobectomy. We detected the risk factors and the relationship between complication and prognosis.

## Introduction

Lung cancer is the most common cancer worldwide and a leading cause of cancer mortality. Surgical complete resection can contribute to a favorable prognosis, especially for early‐stage non‐small cell lung cancer (NSCLC).^1^, ^2^ Recently, video‐assisted thoracoscopic surgery (VATS) was recommended as a safe and effective minimally invasive surgical approach for early‐stage NSCLC.^3^ In a recent randomized controlled trial, VATS reduced early and late fatal pulmonary complications and resulted in less impairment to quality of life.^4^ However, the incidence of postoperative complications for patients who underwent a lobectomy with the VATS approach has been reported as 9%–37%, with a 30‐day postoperative mortality rate of up to 3%.^5^, ^6^, ^7^, ^8^, ^9^, ^10^

Complications prolong hospitalization, increase medical costs, impair the patient's quality of life, and place a burden on medical staff.^11^, ^12^ It is therefore important to assess the potential for postoperative complications before surgery and prepare an appropriate plan to reduce perioperative mortality and morbidity. This requires the risk factors for complications after lung cancer operation to be clearly established.

In this study, therefore, we retrospectively analyzed the surgical complications associated with complete single‐lobe thoracoscopic lobectomy for clinical stage I NSCLC patients and evaluated multiple factors that potentially contributed to these, including sarcopenia (the depletion of skeletal muscle). It has been reported that complications were associated with poorer oncological outcomes, although this finding is controversial^13^, ^14^; we therefore examined the impact of postoperative complications on disease‐free survival (DFS) as an oncological outcome.

## Methods

### Patients

This study retrospectively reviewed the data of 353 consecutive patients who underwent curative complete single‐lobe thoracoscopic lobectomies for clinical stage I NSCLC at our institution between April 2013 and March 2018. These data had been collected prospectively in a database registered and approved by the Review Board of the School of Medicine, Jikei University (approval number: 30–375 [9396]). The following exclusion criteria were applied: clinical stage II or III cancer, incomplete video‐assisted thoracotomy, incomplete resection, bilobectomy, incomplete data and no computed tomography (CT) image at the level of the third lumbar vertebra. The involvement of the mediastinal lymph node was assessed without using CT enlargement or hyperactivity in a positron emission tomography (PET)‐CT scan.

### Data

We collected data on the following patient characteristics: age, sex, smoking history, body mass index (BMI), Charlson Comorbidity Index score (CCI), diabetes mellitus, sarcopenia, and spirometry test results, as well as the preoperative blood examination values of hemoglobin, cholinesterase, albumin, and creatinine. We also recorded the prognostic nutritional index, calculated as 10 × serum albumin (g/dL) + 0.005 × total lymphocyte count (cells/mm^3^).^15^ Operative outcomes included pleural adhesion but excluded a pleural adhesion band, mediastinal lymph node dissection, conversion to thoracotomy, operative time, and surgical blood loss. The data recorded for the postoperative course included chest tube duration and length of postoperative hospitalization. The histological assessment of adenocarcinoma or nonadenocarcinoma was performed according to the World Health Organization histological classification. The pathological TNM determination was based on the criteria in the eighth edition of the Union for International Cancer Control Classification.^16^, ^17^

### Postoperative complications and outcome measure

Postoperative complications included prolonged air leak (chest tube duration ≥7 days), atrial arrhythmia, pneumonia, hypoxia needing home oxygen therapy, empyema, atelectasis, chylothorax, pulmonary embolism, and cerebrovascular disease.

The oncological outcome was represented by the DFS, defined as the interval from the date of surgery to the date of cancer recurrence, or the final follow‐up. Most patients were followed‐up every three or four months for the first three years postoperatively and every four to six months thereafter. Screening examination included tumor markers, chest and abdominal CT, cerebellar magnetic resonance imaging, bone scintigraphy, and PET‐CT on demand. Three‐year DFS was compared between groups of patients with and without postoperative complications.

### Definition of sarcopenia

Sarcopenia is the loss of muscle mass and function. Some reports have defined sarcopenia using the psoas muscle index (PMI) calculated from CT imaging at the level of the third lumbar vertebra.^18^, ^19^ However, there is no agreed cutoff value that defines sarcopenia, with authors of the reports each establishing their own definition. We used a Synapse Vincent three‐dimensional analysis system (Fujifilm Medical Co., Ltd., Tokyo, Japan) as a workstation of electronic chart. We were able to evaluate previous CT images after March 2013 using our electronic chart. We measured the bilateral psoas muscle by manually tracing a CT image acquired within two months before surgery. The PMI was then calculated as the cross‐sectional area of the bilateral psoas muscle divided by the square of the patient's height. The PMI cutoff value for sarcopenia was defined as the median value –1 standard deviation: 4.61 cm^2^/m^2^ for men, and 3.26 cm^2^/m^2^ for women.

### Statistical analysis

Statistical analysis was performed using SPSS version 21.0 software (IBM Corp., Armonk, NY, USA). A two‐sided *P*‐value <0.05 was considered to be statistically significant. Spearman's rank correlation coefficients were calculated to assess the correlations between the data parameters and the complications, and the data parameters and sarcopenia in males and females. PMI between males and females were assessed by Mann–Whitney U test as appropriate. Parameters with *P* < 0.05 in the univariate analysis were selected for inclusion in multivariable logistic regression analysis. DFS and cancer‐specific survival were evaluated with cumulative survival probability using the Kaplan–Meier method, and differences in survivor function between patient groups were assessed using the log‐rank test. Parameters in the univariate analyses with *P* < 0.05 were selected for inclusion in multivariate analysis using a Cox proportional hazard model.

## Results

### Patient characteristics

During the study period, 353 patients underwent lobectomy for primary lung cancer surgery at our institution. Of these, 173 cases (49%) met the criteria and were included in the study. The median follow‐up period was 31 months (range, 2–69 months). The patients' median age was 68 years (range, 31–89 years); there were 98 men and 75 women. The median operation time was 239 min (range: 140–531 min) and the median intraoperative blood loss was 40 mL (range: 0–1250 mL). Two cases (1.2%) underwent conversion to thoracotomy, both because of vascular injury. The median chest tube duration was three days (range, 1–25 days), and the median postoperative hospital stay was seven days (range, 4–40 days). The main pathological type was adenocarcinoma (*n* = 143, 83%). Pathological lymph node metastasis was diagnosed in 19 cases (11%). There were no perioperative or 30‐day postoperative deaths.

### Postoperative complications and risk factors

Postoperative complications developed in 38 (22%) patients, including 21 with prolonged air leak, seven with arrhythmia, three with bacterial pneumonia, three with delayed air leak needing re‐drainage, two with atelectasis, one with empyema, one with hypoxia needing domiciliary oxygen therapy, one with pulmonary embolism, one with chylothorax, one with cardiac decompensation, one with cerebrovascular disease, two with hepatic dysfunction, and one with restlessness.

Table 1 summarizes the results of the univariate analysis comparing clinicopathological factors between the patients that experienced postoperative complications and the remaining patients. The statistically significant risk factors for postoperative complications were elderly age (r = 0.29, *P* < 0.01), male sex (r = 0.19, *P* = 0.013), higher CCI (r = 0.20, *P* = 0.01), lower prognostic nutritional index score (r = −0.16, *P* = 0.043), pleural adhesion (r = 0.22, *P* = 0.005), operative time ≥ 5 hours (r = 0.17, *P* = 0.026), nonadenocarcinoma cancer (r = 0.20, *P* = 0.01), and larger solid component size (r = 0.19, *P* = 0.015). Complications were associated with both a prolonged chest tube drainage period and longer postoperative hospitalization (r = 0.43/0.45, both *P* < 0.01). There were also near‐significant associations between postoperative complication and smoking index ≥600 (r = 0.07, *P* = 0.364), and lower albumin (r = −0.14, *P* = 0.060). There were no significant differences between the groups for sarcopenia, poor spirometry values, or for surgical findings other than pleural adhesion. The variables with *P* < 0.05 in the univariate analysis were included in a multivariable logistic regression analysis model (Table 2). This confirmed that age ≥ 75 years (odds ratio [OR] 3.55, CI 95% 1.53–8.16; *P* = 0.03) and pleural adhesion (OR 3.55, CI 95% 1.33–9.45; *P* = 0.013) were independent risk factors for postoperative complications.

**Table 1:** Univariate analysis of potential predictors of postoperative complications

	Overall (*n* = 173)	No complication (*n* = 135)	Complication (*n* = 38)		
Variables	*n* (%), median ± SD	*n* (%), median ± SD	*n* (%), median ± SD	R‐vale	P‐value
Age (years)	68 ± 10.1	67 ± 9.7	74 ± 10.1	0.29	<0.01
Male sex	98 (56.6%)	70 (51.9%)	28 (73.7%)	0.19	0.013
Smoking index ≥600	66 (38.2%)	49 (36.3%)	17 (44.7%)	0.07	0.364
CCI score	2 ± 1.4	1 ± 1.4	2 ± 1.5	0.20	0.010
Albumin (g/dL)	4 ± 0.5	4 ± 0.51	4 ± 0.46	−0.14	0.060
FEV1.0% <70%	46 (26.6%)	34 (25.1%)	12 (31.6%)	0.08	0.306
Sarcopenia	59 (34.1%)	52 (38.5%)	7 (18.4%)	0.03	0.734
PNI	50 ± 5.2	51 ± 5.2	49 ± 5	−0.16	0.043
Operative findings					
Pleural adhesion	33 (19.1%)	22 (16.3%)	11 (28.9%)	0.22	0.005
Mediastinal lymph node dissection	91 (52.6%)	73 (54.1%)	18 (47.4%)	−0.06	0.464
Conversion to thoracotomy	3 (1.7%)	3 (2.2%)	0 (0%)	−0.06	0.450
Operative time ≥ 5 h	30 (17.3%)	19 (14.1%)	11 (28.9%)	0.17	0.026
Amount of bleeding (>100 mL)	47 (27.2%)	34 (25.2%)	13 (34.2%)	0.10	0.212
Postoperative course					
Chest tube duration (day)	3 ± 3.1	3 ± 1.3	7 ± 5.2	0.43	<0.01
Postoperative hospitalization (day)	7 ± 5	7 ± 3.1	10 ± 8.3	0.45	<0.01
Pathological findings					
Nonadenocarcinoma	30 (17.3%)	18 (13.3%)	12 (31.6%)	0.20	0.010
Solid component diameter (SCD)	12 ± 10	12 ± 9.6	17 ± 10.6	0.19	0.015
Lymph node metastasis	19 (11%)	14 (10.4%)	5 (13.2%)	0.03	0.662

CCI, Charlson Comorbidity Index; PNI, prognostic nutritional index; FEV1.0%, forced expiratory volume in 1 second as a percent of forced vital capacity.

**Table 2:** Multiple logistic regression analysis of potential predictors of postoperative complications

Variables	OR	CI 95%	*P*‐value
Male sex	1.53	0.63–3.75	0.346
Age ≥ 75 years	3.55	1.53–8.16	0.030
CCI ≥3	1.33	0.72–4.61	0.213
Pleural adhesion	3.55	1.33–9.45	0.013
Nonadenocarcinoma	2.33	0.93–5.86	0.078
Solid component diameter > 20 mm	1.50	0.69–3.70	0.381

OR, odds ratio; CI, confidence interval; CCI, Charlson Comorbidity Index.

### Sarcopenia

Sarcopenia was identified in 28 (29%) of the male patients and 30 (40%) of the female patients. Table 3 summarizes the subgroup analysis of differences between the patients with and without sarcopenia. The median PMI was significantly higher for the men than the women (4.99 ± 1.51 [range, 2.32–10.34] vs. 3.5 ± 0.99 [range, 1.54–6.23] cm^2^/m^2^, *P* < 0.01). Among the male patients, PMI was significantly associated with BMI (r = −0.27, *P* = 0.008). For both sexes, anemia (defined as a hemoglobin level below the minimal reference value for that sex) was significantly associated with sarcopenia (male/female: r = 0.33/0.23, *P* = 0.001/0.047). There was also a near‐significant association between advanced age and sarcopenia (male/female: r = −0.19/−0.22, *P* = 0.068/0.088).

**Table 3:** Comparison of characteristics between the patients with and without sarcopenia

	Male (*n* = 98)				Female (*n* = 75)			
	Without sarcopenia (*n* = 70)	Sarcopenia (*n* = 28)			Without sarcopenia (*n* = 45)	Sarcopenia (*n* = 30)		
Variables	*n* (%), Median ± SD	*n* (%), Median ± SD	R‐value	*P*‐value	*n* (%), Median ± SD	*n* (%), Median ± SD	R‐value	*P*‐value
Age (years)	69 ± 9.8	72 ± 8.7	0.19	0.068	69 ± 10.8	64 ± 9.7	−0.22	0.088
Smoking history	57 (81.4%)	24 (85.7%)	0.11	0.301	8 (17.8%)	10 (33.3%)	−0.01	0.949
BMI (kg/m^2^)	23.1 ± 3.3	21.5 ± 2.8	−0.27	0.008	20.8 ± 3.1	20.5 ± 3.3	−0.09	0.477
CCI score	2 ± 1.5	2 ± 1.9	0.14	0.178	1 ± 1	1 ± 1.1	−0.06	0.651
DM	11 (15.7%)	4 (14.3%)	0.11	0.850	1 (2.2%)	3 (10%)	0.17	0.157
Blood test								
Hemoglobin (g/dL)	14.6 ± 1.7	13.3 ± 1.3	−0.33	0.001	13.1 ± 1.1	13.6 ± 1	0.23	0.047
Cholinesterase (U/L)	322 ± 72.2	306 ± 81	−0.12	0.248	326 ± 70.5	320 ± 151	0.06	0.613
Albumin (g/dL)	4 ± 0.5	4 ± 0.5	−0.04	0.708	4 ± 0.5	4 ± 0.5	0.17	0.135
Creatinine (mg/dL)	0.91 ± 1.7	0.89 ± 2.3	−0.06	0.543	0.67 ± 0.3	0.66 ± 1.3	−0.10	0.426
PNI	50 ± 5.7	49 ± 4.9	−0.09	0.366	51 ± 4.3	51 ± 5.1	0.04	0.718
Spirometry test								
%VC <80%	1 (1.4%)	1 (3.6%)	0.07	0.507	0 (0%)	2 (6.7%)	0.26	0.058
FEV1.0% <70%	25 (35.7%)	12 (42.9%)	0.11	0.326	8 (%)	2 (6.7%)	−0.16	0.205
Pathology								
Nonadenocarcinoma	17 (24.3%)	8 (28.6%)	0.04	0.692	4 (8.9%)	1 (3.3%)	−0.12	0.327
Lymph node metastasis	5 (7.1%)	3 (10.7%)	0.06	0.593	7 (15.6%)	4 (13.3%)	0.02	0.865

BMI, body mass index; CCI, Charlson Comorbidity Index; DM, diabetes mellitus; PNI, prognostic nutritional index; %VC, percent of vital capacity; FEV1.0%, forced expiratory volume in 1 second as a percent of forced vital capacity.

### Prognostic significance of postoperative complications

**Figure 1:**
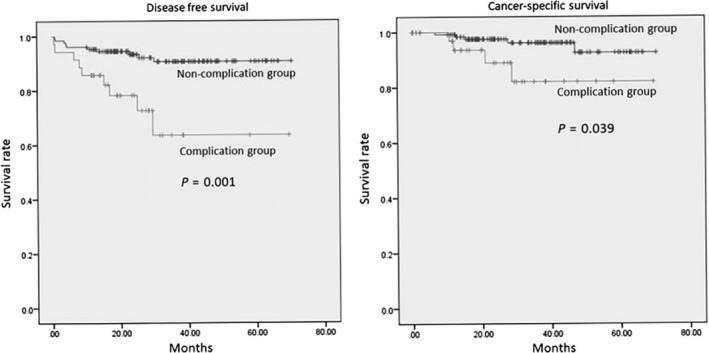
Kaplen‐Meier curves for disease‐free‐survival (**a**) and cancer‐specific survival (**b**).

At the median follow‐up duration of 31 months, 20 (12%) of the patients had experienced recurrence. Three‐year DFS was 91% for the noncomplication group and 64% for the complication group; this difference was significant (*P* = 0.001, log‐rank test). The three‐year cancer‐specific survival was 96% for the noncomplication group and 82% for the complication group; this difference was significant (*P* = 0.039, log‐rank test) (Fig. 1).

Table 4 summarizes the univariate analysis of DFS and clinicopathological parameters, including postoperative complications. Poor DFS was significantly associated with CCI ≥ 3, albumin <3.9 g/dL, prognostic nutritional index < 40, nonadenocarcinoma cancer, lymph node metastasis, solid component diameter > 20 mm, and postoperative complications (all *P* < 0.05, log‐rank test).

**Table 4:** Univariate analysis of disease‐free survival and clinicopathological characteristics (log‐rank test)

Variables	*n* (%)	*P*‐value
Age ≥ 75 years	43 (24.9%)	0.719
Male sex	30 (17.3%)	0.331
Smoking history	106 (61.3%)	0.181
BMI <18.5 (kg/m^2^)	27 (15.6%)	0.099
CCI ≥3	30 (17.3%)	0.046
Albumin <3.9 g/dL	24 (13.9%)	0.009
FEV1.0% <70%	46 (26.6%)	0.154
Sarcopenia	58 (33.5%)	0.932
PNI < 40	7 (4%)	0.043
Nonadenocarcinoma	30 (17.3%)	0.569
Solid component diameter > 20 mm	43 (24.9%)	<0.01
Lymph node metastasis	19 (11%)	<0.01
Postoperative complication	38 (22%)	0.003

BMI, body mass index; CCI, Charlson Comorbidity Index; FEV1.0%, forced expiratory volume in 1 second as a percent of forced vital capacity; PNI, prognostic nutritional index.

The parameters with *P* < 0.05 in the univariate analysis were included in a Cox regression survival analysis model. Table 5 summarizes the results which showed that the following were independent risk factors for poor prognosis: albumin <3.9 g/dL (OR 9.68, CI 95% 1.92–17.61; *P* = 0.002), lymph node metastasis (OR 19.32, CI 95% 3.82–33.00; *P* < 0.01), solid component diameter >20 mm (OR 5.94, CI 95% 1.28–9.39; *P* = 0.015), and postoperative complication (OR 6.70, CI 95% 1.40–11.50; *P* = 0.010).

**Table 5:** Cox regression survival analysis for disease‐free survival

Variables	OR	CI 95%	*P*‐value
CCI ≥3	1.47	0.64–6.53	0.225
Albumin <3.9 g/dL	9.68	1.92–17.61	0.002
PNI < 40	0.54	0.31–13.39	0.461
Lymph node metastasis	19.32	3.82–33.00	<0.01
Solid component diameter > 20 mm	5.94	1.28–9.39	0.015
Postoperative complication	6.70	1.40–11.50	0.010

OR, odds ratio; CI, confidence interval; CCI, Charlson Comorbidity Index; PNI, prognostic nutritional index.

## Discussion

This study reviewed the multiple risk factors related to postoperative complications after single‐lobe thoracoscopic lobectomy for clinical stage I NSCLC. Thoracoscopic surgery was recently recommended as a minimally invasive surgical approach for early‐stage NSCLC, because it exhibited equivalent oncologic outcomes to thoracotomy but with lower overall complication rates, shorter hospital stays, and better postoperative quality of life.^3^, ^4^ However, there is a potential risk with thoracoscopic lobectomy that unexpected emergency thoracotomy may be required because of a major intraoperative complication. This has been reported to occur in 2.5%–23% of cases, and is associated with several complications, including atrial fibrillation, prolonged air leak, the need for blood transfusion, sputum retention, and acute kidney failure.^20^, ^21^, ^22^, ^23^ The rate of conversion to thoracotomy (1.2%) in our study was lower than that of previous reports, perhaps because of selection bias in the patients. We were unable to study the association between conversion to thoracotomy and morbidity because only two patients in our series required conversion. However, the results showed that the safety of thoracoscopic lobectomies for early‐stage lung cancer at our institution was similar to that reported in other studies.

Lung cancer is typically found in elderly people, and advanced age increases the risk of complications with lung cancer operations. Age has been shown to be an important risk factor for morbidity and mortality after lung resection,^24^, ^25^, ^26^, ^27^ with patients aged ≥75 years significantly more likely to experience postoperative complications.^26^, ^28^ Many other risk factors have been significantly associated with postoperative complications, including male sex, a smoking history, poor immunonutritional condition, and low BMI. Zhang *et al*. reports that CCI did not impact on adverse postoperative outcomes following thoracoscopic lobectomy.^29^ Our univariate analysis revealed higher CCI was statistically significant risk factor for postoperative complications, but it was not related on multivariable analysis (OR 1.33, CI 95% 0.48–4.08; p = 0.619). Surgeons have questioned how to define “high risk” for lung resection patients. In the present study, advanced age was one of the strongest preoperative independent predictors of complications following thoracoscopic lobectomy for clinical stage I NSCLC patients. We consider that the minimally invasive thoracoscopic lobectomy procedure might tolerate any other clinical parameters, excluding advanced age.

Pleural adhesion is one of the main reasons for the conversion from a thoracoscopic approach to open thoracotomy, and many less experienced surgeons consider the VATS procedure to be contraindicated in cases of pleural adhesion. Pleural adhesion has been reported to be a statistically significant independent risk factor, not only for conversion to thoracotomy, but also for higher morbidity.^30^, ^31^, ^32^ Pleural adhesions can increase the incidence of prolonged air leak, pneumonia, and atelectasis, and can prolong postoperative hospitalization.^32^ The results of the present study showed that pleural adhesion was the most important risk factor for morbidity, as a common outcome. Because of the complexity of the thoracoscopic procedure for a difficult pleural adhesion and the risk of intraoperative lung injury, we suggest that the procedure is performed by an experienced surgeon, and that the surgeon should not go beyond safe adhesiolysis.

In this study, we evaluated the risk factors associated with poor DFS in a multivariate analysis. This showed that albumin <3.9 g/dL, lymph node metastasis, solid component diameter > 20 mm, and postoperative complications were statistically significant independent risk factors. It is indisputable that advanced cancer is associated with a poorer prognosis. Fan *et al*. examined whether hypoalbuminemia was associated with worse survival in 2988 patients with operable and inoperable lung cancer and concluded that hypoalbuminemia had potential prognostic value besides systemic inflammatory response.^33^ Rivadeneira *et al*. noted that hypoalbuminemia is associated with an impaired immune response through macrophage activation.^34^ The present study showed that hypoalbuminemia was an independent prognostic factor. An association between postoperative complications and oncological poor prognosis has been reported, although this has been controversial.^13^, ^14^ Postoperative complications have been reported to be an independent factor associated with poorer prognosis following VATS lobectomy for NSCLC patients or those with stage I NSCLC.^10^, ^14^ Wang *et al*. suggested that postoperative complications may cause inflammatory and immunosuppressive states associated with poorer long‐term outcomes.^14^ In our study, highly malignant tumors with larger solid component were associated with postoperative complications in univariate analysis, but they were not independent risk factors for postoperative complications in multivariable logistic regression analysis. Additionally, Cox regression survival analysis showed lower albumin (<3.9 g/dL), lymph node metastasis, larger solid component diameter (>20 mm), and postoperative complications were independently associated with poor prognosis. We therefore suggest that postoperative complications exert an independent impact on poor immunonutritional and health conditions, which may result in a poorer oncological prognosis.

Several studies have reported that sarcopenia was associated with poor prognosis in lung cancer, but most of these included patients with advanced stage cancer.^35^, ^36^, ^37^, ^38^, ^39^ According to a rare report about sarcopenia in stage I NSCLC patients, sarcopenia was associated with poor prognosis in men but was not associated with postoperative complications.^40^ Another report, which defined sarcopenia using the erector spinae and pectoralis muscles, suggested that sarcopenia was not associated with the overall complication rate after lobectomies (mostly performed using the VATS approach).^41^ In our study, sarcopenia was not a risk factor for postoperative complications or shorter DFS after thoracoscopic lobectomies for clinical stage I NSCLC.

### Limitations

Several limitations need consideration. First, our study was a retrospective observational study and more than half of the patients initially assessed were excluded; it is therefore possible that the inevitable bias associated with the study design may have affected our analysis. Second, we evaluated poor spirometry outcome and postoperative complication, but data on emphysema, and interstitial pneumonia were not available for analysis in our data set. In addition, the follow‐up period was relatively short (median, 31 months) for the analysis of the impact on DFS. The three‐ and five‐year DFS rates did not differ between the groups, with and without complications. Further prospective studies with more accurate data are necessary to confirm our findings.

In conclusion, advanced age and pleural adhesion were independent risk factors for complications following complete single‐lobe thoracoscopic lobectomies for clinical stage I NSCLC. Postoperative complications were statistically associated with poor prognosis. Surgical teams should ensure an experienced surgeon leads the procedure for patients at high risk to avoid prolonged postoperative hospitalization and a possible poor prognosis.
